# Brain banking for research: can we achieve efficient banking?

**DOI:** 10.3389/fneur.2026.1771564

**Published:** 2026-01-28

**Authors:** Irina Alafuzoff, Björn Ersson, Sylwia Libard

**Affiliations:** 1 Department of Pathology, Uppsala University Hospital, Uppsala, Sweden; 2 Department of Immunology, Genetics and Pathology, Uppsala University, Uppsala, Sweden

**Keywords:** ageing related neurodegeneration, autopsy, biobank, formalin fixed paraffin imbedded (FFPI), frozen tissue, neuropathological assessment

## Abstract

For many years, clinical and surgical pathologists have searched for explanations as to why human tissue is altered, why mass lesions are observed or why the function of an organ is disrupted. One way to answer these questions has been to assess tissue with the naked eye and/or using light- or electron-microscopes, and to compare what is seen in the lesioned tissue with what is seen in normal tissue. Lesions have then been described and named primarily by clinical and surgical pathologists. The practice of sampling tissue for assessment has led to the creation of biobanks—archives of tissue samples that have been analysed and stored for future use. With appropriate ethical approval, these tissue archives have been used extensively for research; over the past decades, our knowledge of various tissue alterations has increased almost exponentially. Below, we have briefly summarised some aspects of work carried out in the field of pathology and its limitations, with particular emphasis on brain pathology.

## Introduction

For many years, pathologists, after receiving a tissue sample from surgeons, have trimmed the sample, placed it in formalin for fixation and subsequently embedded it in paraffin, producing what is known as formalin-fixed paraffin-embedded (FFPE) tissue blocks. In the next step, sections are produced and stained with histological stains. These sections are assessed by a clinical or surgical pathologist, and a diagnosis is assigned according to the criteria valid at the time.

The FFPE blocks and slides have been, and continue to be, stored in archives, i.e., “biobanks.” When needed, for example, in cases of tumour recurrence or when a relative develops a similar disease, the FFPE blocks or stained slides are retrieved, reassessed and further analysed. The general policy is that patients consent to the storage of their samples in archives for future medical use. If a patient declines storage (rarely), the samples are, in some countries, discarded. Laboratories do not own these samples; rather, they serve as custodians of the archives. Such tissue archives have been established across the world in most hospitals, particularly in university hospitals with clinical and surgical pathology departments. Information regarding the blocks, slides, stains performed and diagnoses assigned is stored in continuously growing databases, i.e., the Laboratory Information Systems. In university hospitals in most countries, tissue blocks have been stored for several decades. All of this has been carried out for the benefit of patients.

Over the past decades, in addition to the FFPE material, frozen tissue samples have also been collected and stored. The practice of sampling frozen tissue was driven by the insight that some techniques cannot be applied to fixed tissue. Epitopes targeted during immunohistochemistry (IHC) to visualise proteins are altered during a long fixation time, as formalin is transformed into formic acid. Formalin fixation also damages DNA/RNA strands, altering the outcome of genetic analyses and thus influencing the interpretation of results (1–3).

The archives contain numerous FFPE tissue blocks, and researchers have worked intensively to develop techniques that facilitate the assessment of genetic alterations in FFPE material. These techniques have been available for some time and are currently used routinely, making them a common mode of practice (4).

Interestingly, some years ago, tissue storage was taken one step forward in the U-CAN project in Sweden. Adult patients undergoing surgery for certain tumours consented not only to having their surgically removed tissue stored as both FFPE and frozen samples for research, but also to the inclusion of a substantial amount of clinical information in the associated database (5).

Similarly, tissue samples have also been obtained, to some extent, from various organs, including the brain, at autopsy for diagnostic purposes. As with surgical samples, FFPE blocks and stained tissue sections have been prepared and stored with the consent of a relative. Thus, biobanking has been ongoing for many years as part of the routine work of clinical and surgical pathology departments.

## Biobanking in the field of surgical pathology

It is standard practice for a tissue sample to be sent to the pathology department when a subject is operated on for a lesion in one of the peripheral organs. The lesion is often a tumour, but various other tissue alterations that cannot be defined only based on gross examination—for example, reactive changes or various types of inflammation—are also sent to the surgical pathology department for characterisation. These samples are assessed in order to assign a diagnosis that will guide the choice of treatment. With ethical approval, researchers have had the opportunity to request tissue of interest for their research purposes. This research activity has led to the identification of various protein and/or genetic alterations, which, in turn, has led to what is today referred to as precision therapy. Some tumours are sensitive to radiotherapy, whereas others respond better to specific cytostatic agents or immunotherapies. Survival times in tumour cases have changed dramatically during the past few decades thanks to the development of new therapies; some diseases can now be completely eradicated, with the patient being cured. This progress is the result of using archived material: studying human tissue, examining the alterations that are seen in the tissue, and implementing various assessment techniques.

One of the major challenges in studying human tissue is the heterogenous case selection, which includes a wide range of variables that must be controlled for, including, among others, gender, age and various lifestyle factors. Thus, to interpret the obtained results reliably, the original cohort needs to be sufficiently large to allow the identification of a smaller, but still relatively large, homogenous study cohort.

To facilitate the assessment of numerous samples, a technique was created: building a tissue microarray (TMA) block, where core samples of FFPE material of interest, each measuring a few millimetres in diameter, are incorporated into one paraffin block (6). With the help of the TMA technique, protein profiles in “normal” tissue have been examined, leading to the creation of the Human Protein Atlas (HPA), which is available online (7). Today, researchers can request TMAs from certain “biobanks” containing samples from a certain type of tumour, for example, one occurring only in females within a certain age range and under certain circumstances.

## Biobanking in the field of brain diseases

Surgical samples of brain tissue are also obtained when imaging reveals a mass change, or in cases where surgery is curative, such as in some forms of epilepsy. Thus, as with peripheral tissue, archives within pathology departments contain tissue samples from brain tumours. These have been studied in detail, and treatment strategies have evolved as knowledge regarding the characteristics of tumours has increased.

Obtaining samples from the brain in cases of neurological and/or psychiatric diseases, however, is complicated. It is generally considered unethical to take a brain sample *in vivo* when no defined mass lesion is present. The closest available approach is to obtain a sample of cerebrospinal fluid, which is currently the method of choice when infectious or inflammatory diseases are suspected. In some countries, cerebrospinal fluid has also been assessed in subjects with neurodegenerative diseases (8, 9).

The only available option, both historically and today, is to assess the brains of subjects with neurological or psychiatric complaints after they have died. This approach, neuropathological (NP) assessment of post mortem (PM) brain tissue, is carried out to identify the pathology considered to be causative of the functional disturbances observed *in vivo*.

## Autopsy service

Surgeons and oncologists require a report from a pathologist describing the altered tissue, i.e., whether the change is reactive or a tumour, how a tumour is characterised, and how the patient should be treated in line with current therapeutic strategies. The primary objective of an autopsy is to determine the cause of death. Autopsies may be performed by a coroner, a medical examiner or a clinical/surgical pathologist. The structure of this system—who performs autopsies—varies from country to country. Referrals for autopsy may be made by a medical doctor, often the primary caregiver, by a coroner or sometimes by a relative. Occasionally, the referral may even be made by the patient while still alive. Both the individual who performs the autopsy and the person who initiates the referral differ from one country to another. Generally, if a patient has severe cardiovascular disease and a clinically verified myocardial infarction, an autopsy is seldom requested. Similarly, when an individual has a known tumour disease that has already been diagnosed, an autopsy is often considered unnecessary. Today, life expectancy in most European countries exceeds 80 years, supported by a wide range of treatments available to cope with chronic diseases. With the available *in vivo* assessment strategies, general practitioners, gerontologists, neurologists, psychiatrists and other clinicians generally presume—based on clinical presentation and various laboratory data—that they can determine which organ has failed and led to the patient’s death, i.e., the primary cause of death. Thus, when individuals reach the age of around 80 years, an autopsy is seldom considered necessary (10). However, some studies have shown discrepancies between clinically presumed causes of death and those established through PM examination (11, 12). In parallel with an ageing population, overall healthcare costs have increased, and the funding allocated for autopsy services has not been sustained. As a result, autopsy services have declined dramatically over the past decades. In most European countries today, autopsies are carried out on only a few per cent of all deceased individuals. Contrary to this development, a review article published in 2025 summarised numerous benefits of clinical autopsies (13).

## Neuropathological assessment

The diagnostic assessment of the body, including the peripheral organs, is generally carried out by a general pathologist, whereas assessment of the brain is performed by a neuropathologist. The general pathologist consults a neuropathologist when needed. Alterations in the brain, except for traumatic lesions, are seldom considered to be the direct cause of death, even when the brain pathology is the primary cause of general deterioration. If a deceased individual referred for autopsy displayed neurological and/or psychiatric symptoms, a NP assessment may be carried out at the request of the relatives, the referring clinician, or on the initiative of the pathologist performing the autopsy. Whether such an assessment is done depends on the availability of a neuropathologist at the centre where the autopsy is performed, or on established routines and financial support for NP consultation.

Quite often, the clinical neurological or psychiatric diagnosis made during life is considered valid even after death and is seldom challenged or deemed to require verification. Today, the primary indications for requesting an NP assessment are a history of recent trauma, suspicion of a genetic disease or a clinical presentation that is highly atypical and impossible to interpret. Rough estimates suggest that, in Europe today, only a very small number of brains from deceased individuals undergo PM assessment.

In contrast, neuropathologists have long advocated for what is referred to as a definite diagnosis, i.e., a diagnosis based on observed pathology rather than solely on clinical presentation. The underlying notion is that the NP assessment should be carried out on as many subjects as possible who present with neurological deficits, in line with how neuropathologists assess brain tumours. This approach is consistent with the efforts of the European Union-supported project “BrainNet Europe,” led by Professor Hans Kretzschmar, which aimed to establish brain-banking through the implementation of standardised, reproducible methods and assessment strategies (14–33).

In conclusion, just as pathologists, surgeons and oncologists do not consider the presence of a mass lesion alone sufficient to guide treatment—requiring instead a detailed characterisation of the lesion to inform an appropriate therapeutic approach—the same logic should be applied for neurological and psychiatric diseases.

## Ageing-related neurodegeneration

In most European countries, current life expectancy exceeds 80 years. Unfortunately, with increasing age, the brain undergoes degeneration (34). As with alterations in most organs, neurodegenerative processes develop over time, and symptoms are observed only once the pathology is substantial. This progression is comparable to coronary heart disease: at an early stage of arteriosclerosis, no symptoms are present; then eventually, mild symptoms such as angina pectoris may occur, and in more severe cases, the heart muscle suffers ischemia due to vascular occlusion. Similarly, neurodegenerative processes often begin without noticeable symptoms, but as the pathology progresses, mild neurological symptoms are noted and may eventually progress to more severe impairments.

Thus, not only the brains of clinically affected individuals but also those of asymptomatic elderly subjects need to be assessed. Numerous studies have indicated that age is the major risk factor for neurodegeneration, and from 60 years of age onwards, most individuals display various brain alterations to differing extents (34).

## Methods used in the field of neuropathology

Some of the lesions in the brain that we are aware of have been visualised and recognised for many years using histochemical stains, including haematoxylin-eosin, Nissl stain, various silver stains, and Luxol fast blue, among others. Since the early 1980s, surgical pathologists and neuropathologists have also used IHC stains to visualise protein alterations in the tissue. One of the caveats with the IHC method is that (a) the protein of interest must be known in advance, and (b) an appropriate antibody must be available to detect that protein. Numerous proteins have been identified in recent years, in line with the work done in the HPA project, and a vast number of commercial antibodies have been produced and are now available for diagnostic use. Noteworthy, compared with tumour tissue, the brain is highly complex, and an additional factor that must be taken into consideration is the neuroanatomical region of interest. It is crucial to determine which brain region and/or cell type is of interest for a certain protein alteration. If an irrelevant neuroanatomical region is assessed for a certain protein, the results might not reflect the reality. Moreover, it has to be taken into account that brain pathology is not as a rule symmetric and thus if alterations are searched for in only one hemisphere the outcome might me misleading or even false. A bilateral sampling is certainly recommended for research purposes (35).

There is a significant difference between the characteristics of surgical samples and those obtained PM. Surgical samples are obtained while the subject is alive, and fixation times are usually short; thus, the characteristics of the tissue are not extensively altered. In contrast, PM tissue may be obtained hours or even several days after death, and fixation times may be long—lasting from a few days to many months or even years. Both the PM delay and fixation time can alter the characteristics of the tissue and may therefore influence the suitability of PM material for different analytical techniques. Optimal brain sampling for research purpose is probably achieved in the setting of registered organized brain banks with enrolment of living donors in a brain donation program following defined operating protocols and national legislations (36).

## Number of neuropathological assessments

When dealing with tumours, a large number of cases have been assessed, and researchers have been able to incorporate numerous cases in their studies using the TMA technique. In contrast, due to the low rate of autopsy activity, assessment of the brain in cases of neurological, and particularly psychiatric, diseases is seldom carried out (37). Furthermore, the brain has a complex neuroanatomical structure, where each region displays its unique vulnerability profile. Consequently, numerous neuroanatomical regions need to be sampled and assessed in each case. The assessment of large numbers of slides can eventually be facilitated using scanned images and artificial intelligence (38). Even in the field of neuropathology, the TMA approach has been suggested as a means of minimising the number of sections that need to be assessed in individual cases (39–42). Whereas 20–40 tumour cases can be incorporated into one TMA, 20–40 neuroanatomical regions from one PM brain can similarly be incorporated into one TMA block. One could eventually also build TMAs combining samples from the same neuroanatomical region obtained from various cases, in line with what is done in tumour research.

There is a naive presumption that clinical neurological or psychiatric diagnoses are sufficiently reliable. Noteworthy, some research reports have indicated that, particularly in the field of neurodegeneration, definite PM diagnoses do not always align with clinically presumed diagnoses (43). Our knowledge of the pathology seen in the ageing brain, or in brains affected by various neurological or psychiatric diseases, remains rather sparse.

If autopsies and NP assessments are not carried out, our understanding of brain pathology in subjects with neurological and psychiatric diseases will never reach the level of insight that has been achieved in the field of tumour pathology. Thus, our knowledge of what should be targeted in the brain, in line with the principles of precision medicine, remains limited. Many questions in the field of neurology and psychiatry that cannot be addressed today could eventually be answered if large study cohorts, including thousands of cases across a range of age groups, were made available for research.

## Personal experience

In Sweden, approximately 12% of all deceased individuals are referred for autopsy, which is a relatively large percentage compared with most other European countries. Most of the subjects referred for autopsy in Sweden are younger males, whereas aged, cognitively impaired females are seldom, if ever, referred (10). In parallel, over the past 15 years, NP assessment has been carried out at our university hospital on approximately 30% of all autopsies. This has been made possible through close collaboration between surgical/clinical pathologists and neuropathologists at our centre. NP assessment has been carried out in all subjects with neurological or psychiatric complaints, in most individuals aged over 80 years at death, and in most cases with an unclear cause of death following gross examination. Noteworthy, the mean age at death in Sweden is currently 85 years for females and 82 years for males. In comparison, the mean age at death in the autopsy cohort undergoing NP assessment is 77 years for females and 75 years for males. The discrepancy of approximately seven to 8 years in age is of major significance, particularly when studying age-related neurodegeneration and must be considered in a research setting.

Overall, most neuropathologists have assessed relatively few PM brains compared with the number of brain tumour cases, and particularly few brains with early stages of neurodegeneration. Based on this scenario, it is legitimate to ask whether we will ever attain the same level of knowledge in neurological and psychiatric diseases as has been achieved in tumour pathology—a level of understanding that is essential for the successful implementation of precision therapies.

## Current brain banks

There are currently two options for obtaining PM brain tissue for research. One approach is to contact a pathology department that provides both autopsy and parallel neuropathology services. From such a department, a researcher may request tissue of interest that is stored in the archives, if an ethical approval is in place. However, the availability, quality and characteristics of the tissue can vary significantly, as the material was originally collected solely for diagnostic purposes.

Secondly, researchers may approach a registered tissue bank—a brain bank whose main purpose is the collection of brain tissue for research. These brain banks sample brain tissue in a relatively standardised manner and, in addition to FFPE material, often collect frozen tissue. Deceased subjects are usually enrolled prior to death, and when they reach the final stage of life, the biobank is informed about the impending event. As soon as possible after death, the brain is removed and tissue samples are taken for both freezing and FFPE processing. Subjects may be enrolled based on a clinical diagnosis, i.e., Alzheimer’s Disease, Parkinson’s Disease or other conditions, or as participants in a defined population-based study (35, 44). Both approaches generally include regular *in vivo* assessments. Furthermore, some neuropathologists have, with ethical approval, established biobanks for their own research, and there are publications incorporating data obtained from several biobanks (45–47).

One of the main pitfalls with all of the above approaches is selection bias: how many individuals from the original defined cohort were referred for autopsy, and how many NP assessments were carried out on members of the original cohort −10, 20, 50%, or even more (48). Another limitation relates to the sampling strategies and assessment methods used, and whether they are reproducible and reliable.

In summary, biobanking of brain tissue for research requires appropriate facilities and competent personnel, as well as an “on call” service. Some brain banks do not assess peripheral tissues and thus do not collect samples from organs such as the heart, kidney, liver, skin or gut—organs that have recently been implicated as being of interest in certain neurodegenerative diseases.

## Discussion

Based on all the aspects described above, there are three substantial limitations to biobanking PM brain tissue for research: the availability of autopsy services, financial support and ethical approval. The frequency of referrals for autopsy and NP assessment is currently alarmingly low. Although there is variation between countries regarding who may refer a deceased individual for autopsy, the common denominator is that the primary objective of an autopsy is to determine the cause of death. In most cases, neurological and psychiatric diseases are not considered the direct cause of death. Generally, individuals with an acceptable clinical assessment and a presumed known cause of death are not referred for autopsy, particularly if they have passed the mean life expectancy of the population. For example, if a subject with a neurological disease is bedridden, develops a deep vein thrombosis, and dies of a pulmonary embolism, the cause of death is considered to be cardiovascular, rather than neurological, and thus no further assessment of the brain is sought. Similarly, when an older, cognitively impaired individual dies of pneumonia, verification of the underlying brain disorder is usually deemed unnecessary. Furthermore, primary caregivers may be reluctant to request an autopsy because of the costs associated with both the autopsy itself and NP assessment. As a result, most individuals suffering from neurological or psychiatric disorders are currently buried with a presumed clinical diagnosis, while a definitive diagnosis based on PM assessment remains uncommon. This is certainly not ideal, as the causes of psychiatric and neurological complaints are highly diverse, and our knowledge of the underlying mechanisms is limited. In line with the above, in 2009, in Sweden a research report indicated that there is a poor agreement between the clinical and the PM obtained definite diagnosis (43).

There are two approaches: a brain only autopsy or a full autopsy including an assessment of the brain. In some cases, a full autopsy is certainly preferable, as the function of peripheral organs may influence the progression of brain diseases. For example, regarding α-synuclein pathology in Lewy body diseases both the function of liver as well as kidneys have been suggested being of interest for the evolvement of brain pathology (49, 50). The costs of autopsy and NP assessment are substantial, and in many countries, the decline in autopsy rates is primarily related to limited healthcare resources. Thus, brain only assessment is probably the preferable mode of action to follow in many cases. Noteworthy, tissue banking for research is not part of routine diagnostic work. Thus, there are costs related to the diagnostic procedures themselves and from the additional sampling of tissue for research purposes. Sampling of brain tissue for research must be carried out shortly after death (on-call activity) and must follow standardised protocols. Multiple regions need to be sampled, not only those directly related to the patient’s clinical symptoms. In addition, fresh frozen tissue must be collected to facilitate techniques that require such material. All of the above require appropriate facilities, trained personnel, the creation of databases, and well organised storage facilities for FFPE blocks, slides and freezers for long-term storage. Thus, the financial costs associated with brain banking are substantial. Costs arise from both clinical diagnostic autopsies and diagnostic NP assessments. It should, however, be kept in mind that the costs of autopsy and NP assessment are relatively modest when compared with those of many various *in vivo* assessment and treatment strategies. Moreover, the rapidly increasing number of aged subjects—many of whom will develop more or less pronounced cognitive impairment—will place an enormous financial burden on healthcare systems if precision medicine is not made available.

In most countries, an autopsy can generally be carried out only after consent has been obtained from the deceased individual’s relatives. It is rare for relatives to deny an autopsy particularly if the cause of a brain dysfunction is not fully established. In contrast, in cases involving trauma or forensic circumstances, an autopsy is often considered obligatory. The collection of frozen tissue from various organs is generally accepted in cases of known or suspected genetic disorders. Noteworthy, the medical community must remain aware that there are still unknown inherited biological characteristics and thus collection of frozen tissue should always be considered. However, the collection of FFPE tissue beyond what is required for diagnostic purposes, as well as the freezing of samples for eventual genetic analyses not part of standard diagnostic procedures, typically requires separate ethical approval.

The final essential requirement is the availability of a neuropathologist—the specialist responsible for executing the precise sampling of the brain and carrying out the diagnostic assessment.

## Conclusion

Tissue archives containing both frozen and FFPE material are essential for the development of precision therapies in the fields of neurology and psychiatry. With an ageing population, there is an urgent need for therapies directed towards defined brain alterations rather than symptoms alone. The brain alterations can only be identified through systematic NP assessment incorporating new methods, including analyses of protein and genetic changes. Thus, a definitive diagnosis of a neurological or psychiatric disease should be obligatory rather than the exception, as it is today. It is naive to assume that all relevant lesions are already known. Quite recently, a new disease entity, i.e., Age-related Limbic-predominant TDP Encephalopathy (LATE), was defined, displaying pathological protein alterations known to us for only about 20 years (51, 52). Consequently, research on human PM brain tissue must be carried out continuously, referrals for autopsy should be actively encouraged and definitive diagnoses based on a NP assessment carried out PM should be considered mandatory particularly for individuals with neurological or psychiatric diseases. Efforts should also be made to support the development of tissue archives in this field, in line with the extensive work that has been done for years in tumour pathology.

## Data Availability

The original contributions presented in the study are included in the article/supplementary material, further inquiries can be directed to the corresponding author.
